# 

**Published:** 1886-10

**Authors:** 


					﻿OCTOBER, 1836-
OLD SERIE^
VoLXXJfr***^
<7 1855 0'
:BIGJL'<UR1S
1 t^lDlIRNABs^R
&8kb<J
Wf .WESTMORELANdW©,
H.VM.M1LLER.M.D.LL ,dJk
JAMES A.GRAY. MJ}.'4
MbUSHED By JAMES RHARRISON&C^j
MP________________SaiLANTA.GA. JM
SUBSCRIPTION: 12.50 A YEAR.
				

